# Can colour vision re-evolve? Variation in the X-linked opsin locus of cathemeral Azara’s owl monkeys (*Aotus azarae azarae*)

**DOI:** 10.1186/s12983-016-0139-z

**Published:** 2016-02-24

**Authors:** N. I. Mundy, N. C. Morningstar, A. L. Baden, E. Fernandez-Duque, V. M. Dávalos, B. J. Bradley

**Affiliations:** Department of Zoology, University of Cambridge, Cambridge, UK; Department of Anthropology, Yale University, New Haven, CT USA; Department of Anthropology, Hunter College of the City University of New York, New York, USA; Department of Anthropology, University of Pennsylvania, Pennsylvania, USA; Facultad de Recursos Naturales, Universidad Nacional de Formosa, Formosa, Argentina; Center for the Advanced Study of Human Paleobiology, The George Washington University, Washington, DC USA

**Keywords:** Aotus, Owl monkey, Platyrrhine, Colour vision, Activity pattern

## Abstract

**Background:**

Do evolutionary specializations lead to evolutionary constraint? This appears plausible, particularly when specialization leads to loss of complex adaptations. In the owl monkey lineage, nocturnality clearly arose from a diurnal ancestor. This behavioural shift was accompanied by morphological changes in the eye and orbit and complete loss of colour vision via missense mutations in the gene encoding the short-wave sensitive visual pigment (SWS opsin). Interestingly, at least one subspecies of owl monkey, Azara’s owl monkey (*Aotus azarae azarae*), has regained activity in daylight. Given that all primate species that are active in daylight, including primarily diurnal species and species that are active during both day and night, have at least dichromatic colour vision, it seems reasonable to propose that dichromacy would be adaptive in *A. a. azarae*. With a disabled SWS opsin, the main avenue available for Azara’s owl monkeys to re-evolve colour vision is via a polymorphism in the intact X-linked opsin locus, which commonly occurs in other New World monkeys. To examine this possibility we assayed variation in the X-linked opsin of *A. a. azarae*, focusing on the three exons (3, 4 and 5) that control spectral sensitivity.

**Results:**

We found low opsin genetic variation on a population level, and no differences at the three main sites that lead to variation in spectral sensitivity in the opsins of other New World monkeys. Two rare alleles with single amino acid variants are segregating in the population, but previous functional studies indicate that these are unlikely to affect spectral sensitivity.

**Conclusions:**

Genetic constraint on the re-evolution of colour vision is likely operating in Azara’s owl monkey, which may affect the niche that this subspecies is able to occupy.

## Background

In evolutionary biology, Dollo’s Law proposes that complex adaptations cannot be reacquired easily once lost [1]. This can therefore lead to a genetic constraint on adaptation if the environment subsequently changes. Although the hypothesis has received much attention and theoretical support, there are few cases in which it has been empirically documented [2]. Owl monkeys (*Aotus*) are the only nocturnal group of anthropoid primates, and they have a suite of visual adaptations for a nocturnal lifestyle including a large eye and orbit size, and a high retinal rod to cone ratio [3]. In common with several other nocturnal primate lineages, owl monkeys lost the capacity for colour vision, having evolved to become monochromatic (completely colour-blind) from a dichromatic ancestor [4]. This occurred as a result of deleterious mutations in the autosomal short wavelength sensitive (SW) opsin locus, leaving a single intact middle wavelength sensitive (MW) opsin on the X chromosome [5].

Unlike other owl monkey species which are strictly nocturnal, the Azara’s owl monkeys (*A. a. azarae*) of Argentina and Paraguay are cathemeral [6], showing considerable amounts of activity both during the dark and light phases of the 24-h cycle [7]. Most of their activity is concentrated at times of limited luminosity (dawn, dusk, and night), but daytime (0900–1800hs) activity is present year round, reaching a maximum (31 %) during the cold winter months and a minimum (13 %) in the summer [7].

All other primate species that are active in daylight, including both primarily diurnal species and species that exhibit substantial periods of activity during both day and night, have either dichromatic or trichromatic colour vision [8, 9] suggesting a pervasive adaptive advantage for colour vision at high luminance levels in primates. Hence, it is hypothesized that the partially diurnal activity pattern of Azara’s owl monkeys has created a selective pressure for dichromacy leading to the re-evolution of adaptive colour vision. One potential mutational mechanism for colour vision to re-evolve would be for the SW opsin to regain functionality, but this has not occurred [10]. A second possibility is that a novel spectrally sensitive allele could evolve at the X-linked MW opsin locus. This change would lead to heterozygous females having two functional opsin alleles, and likely a form of dichromacy, whereas homozygous females and hemizygous males would remain monochromatic. This situation would be analogous to the X-linked opsin polymorphism in most other New World monkeys, except that in these species the SW locus is intact resulting in dichromatic males and heterozygous trichromatic females [8]. Mutations impacting X-linked opsin sensitivity have occurred repeatedly along multiple lineages during primate evolution [11], so it would not be surprising if Azara’s owl monkeys exhibited such a polymorphism. Here we examine this hypothesis in a wild population of Azara’s owl monkeys by assaying sequence variation in the regions of the MW opsin gene that determine spectral sensitivity.

## Results and discussion

We obtained the full coding sequence of exons 3–5 of the X-linked opsin gene for 20 females. There was no amino acid variation at the three key sites (site 180 in exon 3 and sites 277 and 285 in exon 5) that are responsible for the majority of variation in maximal spectral sensitivity (λ_max_) in primates (Table 1). These sites are identical to those in the conspecific *A. a. boliviensis*, whose MW opsin has an experimentally determined λ_max_ of 539 nm [12]. Two rare amino acid variants, D294N and K312R, both in exon 5, were present as heterozygous variants in two individuals (frequency of both variants 5 %), and are likely linked. K312R is a conservative mutation that is not at a site that affects spectral sensitivity. D294N is interesting because it affects spectral sensitivity (reduction in λ_max_) in spider monkeys, but only in the presence of a second variant, Y213D [13]. As Y213D is absent in Azara’s owl monkey it is unlikely that D294N affects spectral sensitivity in this taxon. While the sample size of 40 X chromosomes was modest, it would have easily been large enough to detect allelic variation at the X-linked opsin locus in the majority of New World monkeys, where the frequency of individual alleles is typically 15–50 % (e.g. see [14]).

**Table 1:** X-linked opsin variation in AzaraZs owl monkeys (*Aotus a. azarae*)

			Ex 3		Exon 4			Exon 5		
		N	**180**	229	213	233	**277**	**285**	294	312
*A. a. azarae*	Allele 1	38	A	I	Y	S	Y	A	N	K
	Allele 2	2	A	I	Y	S	Y	A	D	R
*A. a. boliviensis*			A	I	Y	S	Y	A	N	K

Variable sites and sites potentially affecting spectral sensitivity are shown in single letter amino acid code, with the three key sites in bold

Thus, overall we found little evidence of functional variation in the X-linked opsin of Azara’s owl monkey which would lead to dichromatism and the re-emergence of colour vision in heterozygous females. Hence, the two main routes to reacquisition of colour vision, which involve the only opsins expressed in retinal cones in primates - regeneration of the SWS opsin [10], and functional variation in the X-linked opsin (this study) - have not occurred. At intermediate (mesopic) light conditions, rhodopsin expressed in rods may play a role in colour vision [4, 15–17], and this would be interesting to explore in the future, particularly since the high rod density in owl monkeys means that their mesopic range likely extends to higher light levels than in diurnal anthropoid primates. However, rhodopsin cannot contribute to colour vision under the daylight (photopic) conditions which Azara’s owl monkeys experience. Another class of opsins, melanopsins, are expressed in some retinal ganglion cells and function in entraining circadian rhythms and in sensing overall luminance, but it is not yet clear whether they are able to contribute to colour vision [18, 19].

Given the high rod to cone density in the retinas of owl monkeys, it is worth considering whether this is a stronger constraint on the re-evolution of colour vision than the regeneration of relevant opsin variation. For example, in mice that were genetically engineered to express an extra human LW opsin, behavioural evidence for colour vision was rare [20]. However, certain lemurs have behaviourally demonstrated colour vision that relies on cone densities that are similar to those found in *Aotus* [21, 22], arguing against a strong constraint based on cone density. Also, it remains to be determined whether there has been any change in cone abundance in *A. a. azarae*. Another possibility is that spectral separation of a novel X-linked opsin may not be sufficient to provide useful colour vision. Human tritanopes are analogous dichromats that do retain useful colour vision with a ~30 nm separation among the MW and LW pigments [23], although smaller spectral separations would be possible in *Aotus*.

Comparative data strongly suggest that colour vision would be adaptive in Azara’s owl monkey; given that they are primarily frugivorous [24], colour vision would likely aid in food selection. All primates that show substantial activity during the daytime, whether strictly diurnal species or species that are active both at day at night [25] have some form of colour vision (dichromatic or trichromatic) [8, 9, 11]. In contrast, all monochromatic primates apart from Azara’s owl monkey, including other owl monkeys, lorisoids and some cheirogaleid lemurs, are strictly nocturnal. The adaptive advantage of dichromacy over monochromacy for daytime activity is poorly studied, particularly in comparison to advantages of trichromacy over dichromacy, and warrants further investigation.

X-linked opsin changes impacting colour vision have evolved independently in several primate lineages [11], but the time scale and historical contingency of such mutations is unclear. While Azara’s owl monkeys may have diverged from other *Aotus* lineages up to a few million years ago [26], there is considerable uncertainty over when the characteristic periods of diurnal activity evolved in *A. a. azarae*. Azara’s owl monkeys are found exclusively in the seasonally dry forests of the South American Gran Chaco and a recent evaluation of mtDNA diversity in this taxon concluded that the expansion into this area may have been relatively recent, occurring after climatic and geographic processes drained the southernmost Chacoan forests and flatlands of water some 5,000–7,000 years ago [26]. With a mutational target in the X-linked opsin locus of just three major sites to alter spectral sensitivity, and a nuclear mutation rate on the order of 0.5 x 10^−9^/site/year in primates [27], the expected time to acquisition of dichromacy would be long.

In some cases, re-evolution of complex structures has occurred after many millions of years (e.g., the re-acquisition of mandibular teeth in frogs [28]). A plausible explanation is that in these cases the developmental pathways underlying the traits that re-evolve are conserved with other structures that are retained during evolution. Hence the functional protein sequences remain and re-evolution of the trait requires mutations restoring gene expression patterns. In contrast, the visual pigments underlying colour vision in vertebrates do not have pleiotropic functions elsewhere in the body, and this has the effect of making restoration of colour vision difficult. This is because loss of colour vision can be accompanied by loss-of- function mutations in the coding sequence of opsin genes, as occurred in the SWS opsin gene in the *Aotus* lineage, and the SWS pseudogene can then further degenerate by accumulation of extra deleterious mutations. Regeneration of colour vision via the SWS opsin then becomes very unlikely and instead precise mutations in the MW opsin must occur.

## Conclusions

A genetic constraint on the re-evolution of colour vision appears to be present in Azara’s owl monkey in the sense that mutations that could restore colour vision have not yet occurred. The absence of colour vision could potentially influence the niche these monkeys are able to occupy. More broadly, the genus *Aotus* offers an excellent opportunity to examine the phenotypic and genetic mechanisms behind adaptations to gross changes in activity pattern, which is a key ecological trait.

## Methods

### Samples

DNA was derived from blood samples collected from 20 wild females of *Aotus azarae azarae* (17 from 16 different social groups and 3 from solitary individuals) as part of the Owl Monkey Project (*Proyecto Mirikin*á) in the Formosa Province of Argentina [29]. DNA was isolated using QIAamp purification kits (Qiagen). All protocols adhered to the laws of Argentina, the United States and the United Kingdom, and were approved by respective animal care and use committees (University of Pennsylvania: IACUC#2010-801089; Yale University: IACUC#2010–11378).

### Amplification and sequencing

Since Y chromosome opsin pseudogenes are present in *Aotus* [30], we eliminated the possibility of these interfering with genotyping by focusing on females. Samples were amplified at exons 3, 4, and 5 of the X-linked opsin using primers designed from an *Aotus azarae boliviensis* reference sequence (GenBank: AB081277.2). 24 μl PCR reactions consisted of: 1.0 μl template, 10 μM of each primer and 12.5 μl Qiagen HotStarTaq Master Mix. Amplification conditions were as follows: 15 m initial denaturation at 95 °C; 30–35 cycles of 30s at 94 °C, 40s at 61 °C, 60s at 72 °C; and a 7 m final extension at 72 °C. Products were sequenced on both strands at the Yale DNA Analysis Facility (ABI3730). Sequence files were aligned and translated using Geneious (Biomatters).

### Availability of supporting data

Novel DNA sequences have been deposited in Genbank (Accession KU728173).
